# Production of Omega-3 Fatty Acid Concentrates from Common Kilka Oil: Optimization of the Urea Complexation Process

**DOI:** 10.3390/molecules29112430

**Published:** 2024-05-21

**Authors:** Zahra Eskandari, Seyed Fakhreddin Hosseini, Anan Yaghmur

**Affiliations:** 1 Department of Seafood Processing, Faculty of Marine Sciences, Tarbiat Modares University, Noor 46414-356, Iran; shadieskandari0704@gmail.com; 2 Department of Pharmacy, Faculty of Health and Medical Sciences, University of Copenhagen, Universitetsparken 2, 2100 Copenhagen, Denmark; anan.yaghmur@sund.ku.dk

**Keywords:** common kilka oil, docosahexaenoic acid, eicosapentaenoic acid, ω-3 PUFAs concentrates, urea complexation, process optimization

## Abstract

There has been an increase in interest in the application of ω-3 PUFAs, especially EPA and DHA, in the development of various food products owing to their myriad health benefits. However, most fish oils do not contain more than 30% combined levels of EPA and DHA. In this study, through the urea complexation procedure, the production of EPA and DHA concentrate in their free fatty acids (FFAs) form was achieved from an enzymatic oil extracted from common kilka (*Clupeonella cultriventris* caspia). To gain the maximum value of EPA and DHA, the response surface methodology (RSM), which is an effective tool to categorize the level of independent variables onto the responses of an experimental process, was also used. Different variables including the urea–fatty acids (FAs) ratio (in the range of 2–6, *w*/*w*), the temperature of crystallization (in the range of −24–8 °C), and the time of crystallization (in the range of 8–40 h) were investigated by response surface methodology (RSM) for maximizing the EPA and DHA contents. Following the model validation, the levels of the variables at which the maximum desirability function (0.907 score) was obtained for response variables were 5:1 (urea–FAs ratio), −9 °C (the temperature of crystallization), and 24 h (the time of crystallization). Under these optimal conditions, increases of 2.2 and 4.4 times in the EPA and DHA concentrations were observed, respectively, and an increase in the concentrations of EPA and DHA from 5.39 and 13.32% in the crude oil to 12.07 and 58.36% in the ω-3 PUFA concentrates were observed, respectively. These findings indicate that the urea complexation process is efficient at optimizated conditions.

## 1. Introduction

There has been an increase in interest in the application of ω-3 PUFAs in the development of various food products owing to their various health-promoting effects, including their attractiveness in minimizing the risks of various diseases [1]. Among ω-3 PUFAs, α-linolenic acid (18:3 n-3; ALA), eicosapentaenoic acid (20:5 n-3; EPA), and docosahexaenoic acid (22:6 n-3; DHA) are of importance for the human body [2]. Since DHA and EPA are essential fatty acids (FAs) and primarily synthesized in aquatic environments, marine organisms such as fish are known as the main sources of these FAs [3]. EPA and DHA play a significant role in many human-related biological processes, and their deficiency in ω-3 PUFA nutrition may cause various disorders, including cardiovascular diseases, high blood pressure, auto-immunity, depression, and neurological diseases [4]. The National Health Committee of China recommends using ω-3 PUFAs in the treatment of acute COVID-19 cases [5]. It was also reported that intravenously or orally administered ω-3 PUFAs can shorten the period for recovery from COVID-19 [6]. An intake of 250 mg EPA + DHA per day, which is equivalent to at least two servings of fish per week, is advised for minimizing the risks of cardiovascular disease [7]. However, most fish oils do not contain more than 30% combined levels of EPA and DHA [8]. Therefore, there is increasing interest in the production of highly concentrated ω-3 PUFAs.

In the literature, various approaches exist for producing ω-3 PUFAs concentrates; these include low-temperature winterization, enzymatic purification, supercritical fluid extraction, molecular distillation, and urea complexation [9]. Regardless of the concentrating method, the chemical form of the final product should also be taken into account, because the bioavailability of ω-3 PUFAs varies depending on the existing forms, e.g., triacylglycerols (TAGs), ethyl esters (EEs), phospholipids (PLs), or FFAs [10]. PUFAs in the form of FFAs are absorbed more efficiently by the intestine than PUFAs in the form of TAGs or EEs [11]. Among them, urea complexation has been introduced as the easiest and most efficient approach to obtain ω-3 PUFA concentrate in the form of FFAs [4,10]. Initially, fish oil triacylglycerol (TAG) becomes fractionated into FFAs via alkaline hydrolysis utilizing NaOH or KOH solutions. Then, the obtained FFAs are blended using a urea ethanolic solution to form complexes. Among these complexes, saturated (SFA) and monounsaturated (MUFA) fatty acids can be separated through filtration after urea crystallization. The non-urea complexing fraction (NUCF) is then concentrated with PUFAs [9].

To gain the maximum value of EPA and DHA, it is worth considering RSM (response surface methodology), which is an effective correlation method between the experimental parameters and the response of the desired objectives. In the Caspian Sea, the following three small pelagic species are the most abundant fish: clupeids (known as “kilka”), including anchovy (*Clupeonella engrauliformis*), big-eyed kilka (*C. grimmi*), and common kilka (*C. cultriventris* caspia) [12]. Among them, common kilka accounted for nearly 97% of the total kilka catch (19,000 metric tons in 2022) in the southern Caspian Sea (Iran) [13]. Despite its richness in oil and protein, just about 5% of the fish catch is consumed by humans, and the rest is converted into fish oil and fish powder and typically used as food additives in animal feed and aquafeeds. This is because of their small size (~10 cm) and quick internal enzymatic spoilage [14]. Better and more efficient use of this low-cost and nutrient-rich fish can be achieved through highly promising innovative technologies focusing on the production of high-added-value products, including ω-3 PUFA concentrates. Thus, the present study aims to focus on the optimization of the urea complexation process to obtain high-ω-3 PUFA concentrates from common kilka (*C. cultriventris* caspia) oil. We hypothesize that the urea complexation process can effectively enrich the ω-3 PUFAs (DHA and EPA) by applying RSM. The applied RSM approach for optimization investigated the influence of different variables, including the urea-to-FA ratio (*w*/*w*), the temperature of crystallization (°C), and the time of crystallization (h).

## 2. Results and Discussion

### 2.1. Fatty Acid Profile of Initial Fish Oil

The FA composition of the initial kilka oil is presented in Table 1. The two most predominant FAs were oleic acid (C18:1n-9, 33.47%) and palmitic acid (C16:0, 18.23%), followed by C22:6n-3 (DHA, 13.32%), C20:5n-3 (EPA, 5.39%), and C16:1n-7 (palmitoleic acid, 4.07%). A similar FA composition has been previously reported for anchovy [15] and sardine oil [16]. The distribution of FA groups obtained in the present study also showed the following proportions: 25.6% (SFA), 40.7% (MUFA), 24.5% (PUFA), and 20.4% (ω-3 PUFA).

### 2.2. Production of ω-3 PUFA Concentrates

Based on the experimental plan displayed in Table 2, 20 runs were conducted. This study reports on the experimental results gained for the following response variables: R1 (EPA content; %FA in the NUCF), R2 (DHA content; %FA in the NUCF), and R3 (EPA + DHA content; %FA in the NUCF) of the kilka oil concentrate. All three independent variables (A: urea–FA ratio, *w*/*w*; B: the temperature of crystallization, °C; and C: the time of crystallization, h) significantly (*p* < 0.05) affected the obtained responses during the process of urea adduction. The highest amount of EPA (17.32%) was obtained with a urea–FA ratio of 4:1, a crystallization temperature of −8 °C, and a 24 h time of crystallization. For DHA, two variables (i.e., the ratio of urea–FA and the time of crystallization) were the same as for EPA (Table 2). However, it was important to change the crystallization temperature to −24 °C to achieve the highest enrichment with DHA (59.67%). As compared to the DHA content (13.32%) in the initial kilka oil, the optimization of the urea complexation process led to a 4.5-fold increase in the content of DHA in the concentrate. It also led to an increase in the total EPA + DHA amount up to 72.43% in the NUCF (Table 2). Thus, our data demonstrate that the formation of complexes between urea and saturated/mono- or di-unsaturated FAs plays a central role in increasing EPA and DHA concentrations [10]. According to Ratnayake et al. [17], the complete removal of SFAs through urea complexation is most likely impossible, since some of the FAs with relatively short chains do not complex with urea over the process of crystallization.

### 2.3. Influence of the Independent Variables on the Response Variables: Regression Coefficients and Pareto Charts

Table 3 presents the modified quadratic polynomial regression equations for the anticipated models of the following response variables: %EPA (R1), %DHA (R2), and %EPA + DHA (R3). This table displays the predictive second-order polynomial models and the regression coefficients of the three response variables. The obtained multiple regression models also show how the three investigated process variables presented in Table 2 affected the production ω-3 PUFA concentrates via the urea complexation process. Through regression analysis, the obtained determination coefficients (R^2^ parameters) for the R1, R2, and R3 variables were 0.95, 0.94, and 0.93, respectively. Also, in order to evaluate whether the chosen models were appropriate to demonstrate the observed experimental data or not, the lack-of-fit test was used. According to the ANOVA analysis, the obtained *p*-values for the lack-of-fit cases were higher than 0.05. This means that all the predicted models seemed to be sufficient to explain the results acquired for the three response variables (R1, R2, and R3) at a confidence level of 95% (Table 3). Focused on oil from rainbow trout by-products, Pando et al. [18] observed that the regression models for EPA, DHA, and EPA + DHA contents were significant with satisfactory R^2^ values as follows: 0.83, 0.78, and 0.84, respectively. In another study, Zheng et al. [19] also found that the regression models for ω-3 PUFAs from seal oil were very significant (*p* < 0.01) with good values (0.99) of R^2^.

Regarding the EPA content (R1, Table 3), the obtained regression coefficients showed that the linear terms of the urea-to-FA ratio (A), the temperature of crystallization (B), the time of crystallization (C), and the quadratic terms of the urea-to-FA ratio (AA) and the time of crystallization (CC) were significant (*p* < 0.05). For the DHA amount (R2, Table 3), the obtained regression coefficients of the three linear terms (i.e., A, B, and C), and the quadratic terms of the crystallization time (CC) were observed to be significant (*p* ≤ 0.01). This indicates that they can be considered determinants for the DHA content in the final product. Analysis of the regression coefficients of the EPA + DHA amount (R3, Table 3) revealed that the linear terms for the urea-to-FA ratio (A), the temperature of crystallization (B), and the quadratic terms of the urea-to-FA ratio (AA) and the time of crystallization (CC) were also significant in the process of urea adduction (*p* < 0.05). This means that all terms most likely play important roles in determining the content of EPA + DHA in the ω-3 PUFA concentrate. A former study on oil enrichment from by-products of rainbow trout processing revealed that the linear and quadratic terms of the urea-to-FA ratio and the temperature of crystallization for the determination of the total EPA + DHA content were very significant [18]. However, the crystallization time did not affect the process of complexation (*p* > 0.05). In another study on seal oil, Zheng et al. [19] showed that the urea–FA ratio had a significant linear influence and played an important role in determining the ω-3 PUFA (EPA + DPA + DHA) concentration (*p* < 0.01), whereas the crystallization temperature and the crystallization time had a quadratic influence (*p* < 0.05) on the mass fraction of ω-3 PUFAs.

### 2.4. Influence of the Independent Variables on EPA, DHA, and EPA + DHA Amounts of Kilka Concentrate

**Figure 1:**
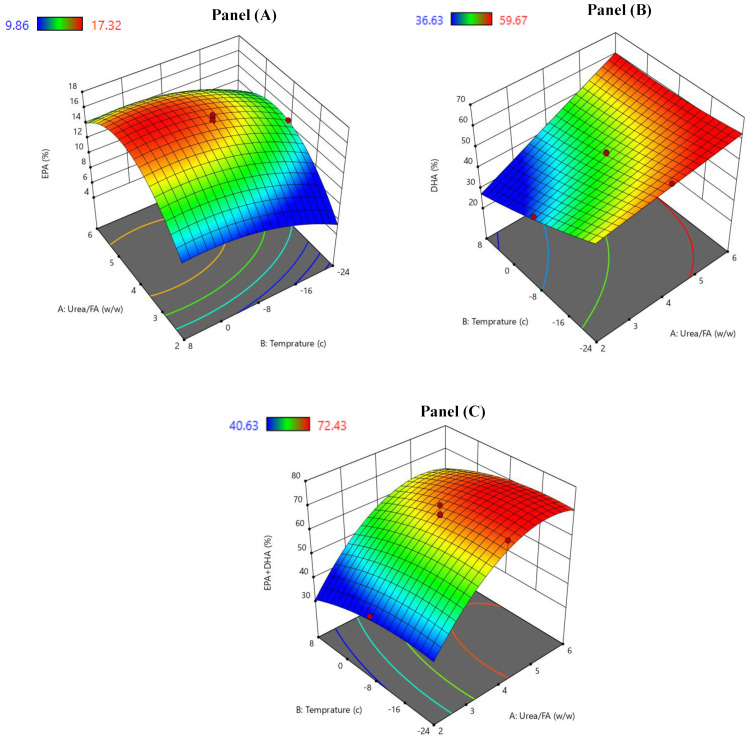
The effects of urea/FA ratio and crystallization temperature on the following responsive variables: (**A**) the EPA content (% of total FAs), (**B**) the DHA content (% of total FAs), and (**C**) the EPA + DHA content (% of total FAs).

The three-dimensional (3D) response surface plots were drawn utilizing the second-order polynomial models (consisting of linear, quadratic, and interaction terms). Subsequently, to analyze the effects of the independent variables on the three response variables, the former variables were placed in different axes (Figure 1A–C). Figure 1A shows the response surface of the urea adduction for the EPA amount. It shows that the EPA amount increased with augmenting the urea-to-FA ratio up to 4.5:1 (*w*/*w*) and then started to decrease (*p* < 0.05) upon a further increase in the urea-to-FA ratio. It can also be seen that an increase in the crystallization temperature (up to 1.5 °C) and time (up to 27 h) was connected with an increase in the EPA amount (*p* < 0.05). In the experimental design for the EPA content, the adjusted R^2^ coefficient also showed a variability of 88.18% (*p* < 0.05) (Table 3). Pando et al. [18] also reported the effect of the urea adduction conditions on modulating the EPA amount. It was reported that increasing both the urea-to-FA ratio and the temperature of crystallization to 3.8:1 and 0 °C, respectively, was related to a remarkable increase in the EPA content [18]. The maximum amount of EPA at intermediate levels was obtained at a 24.4 h crystallization time.

Figure 1B shows the effects of the urea complexation conditions on the DHA content in the ω-3 PUFA concentrates. The response surface of the urea adduction procedure indicates a difference in the behavior when comparing the effects of the urea-to-FA ratio and the temperature of crystallization. Clearly, the urea-to-FA ratio had a positive impact on the DHA content, while the temperature of crystallization showed a negative influence (*p* < 0.05). As indicated by the adjusted R^2^ values shown in Table 3, the fitted model accounted for 87.31% of the variability in the DHA content. Our results are in good accordance with previous research reporting on an inverse relationship between the temperature of crystallization and the urea-to-FA ratio [20,21].

Further, it was interesting to investigate the influence of the investigated two process variables (urea-to-FA ratio and the temperature of crystallization) on the EPA + DHA amount. Figure 1C displays the response surface for the content of EPA + DHA as a result of the urea–FA ratio and crystallization temperature. Clearly, the %EPA + DHA content in the NUCF increased with increasing the urea–FA ratio up to 5:1 (*w*/*w*) and decreasing the crystallization temperature up to −14 °C (*p* < 0.05). In this study, the adjusted R^2^ value revealed a variability of 85.43% (*p* < 0.05) for the EPA + DHA content (Table 3). Our findings are in agreement with those reported by Dovale-Rosabal et al. [20], who found an inverse relation between the temperature of crystallization and the urea-to-FA ratio during the urea complexation process of refined salmon oil.

### 2.5. Process Parameters and Multiple Response Optimization

**Figure 2:**
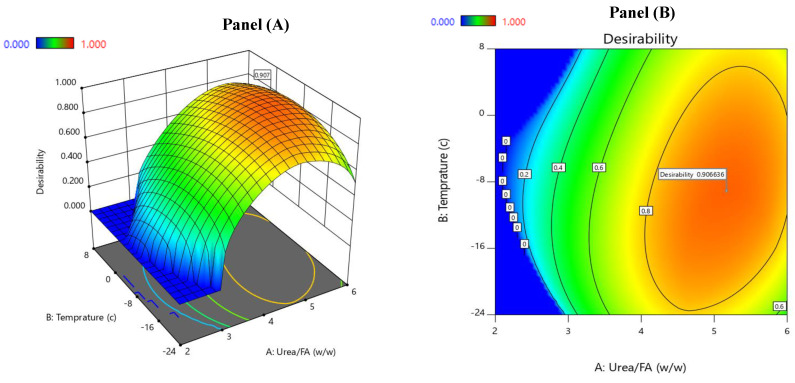
Combination of factors to maximize the desirability function for kilka oil ω-3 PUFA concentrate: (**A**) the desirability function, and (**B**) the contour plot of the estimated response surface.

Table 4 presents the required levels of factors for maximizing the three investigated response parameters (i.e., EPA, DHA, and EPA + DHA) in the ω-3 PUFA concentrates for the denoted region shown in Figure 2A,B. For maximizing the EPA content, it was important to consider the following combination of the urea-to-FA ratio, the temperature of crystallization, and the time of crystallization: 4.67, 1.62 °C, and 27.48 h, respectively. Under these conditions, the predicted optimum value was 17.4% (Table 4, Part a). Regarding the DHA content, a combination of 5.76 (urea-to-FA ratio), −13.35 °C (the temperature of crystallization), and 20.92 h (the time of crystallization) resulted in an optimum predicted value of 60.76% (Table 4, Part a). Subsequently, similar conditions of the process variables were required to achieve the maximal levels of EPA + DHA (73.66%; Table 4, Part a). Here, the highest stationary point was obtained at the following conditions: urea–FA ratio of 5.31, crystallization temperature of −13.97, and 21.52 h crystallization time. These findings were in good agreement with the previous findings of Wanasundara and Shahidi [22]; for EPA + DHA contents, the maximum value of 92.3% was reported at the following combination: a urea–FA ratio of 4.3, a temperature of crystallization of −11 °C, and a 19 h crystallization time. In the ω-3 PUFA concentrates, the presence of a relatively higher DHA content than that of EPA was most likely attributed to the lower tendency of DHA to form complexes with urea. These findings were in good accordance with previous reports on DHA and EPA contents in belly muscle oil from rainbow trout by-products [18]; it was noted that DHA was the most plentiful FA in the NUCF during the urea complexation process.

Table 4 also exhibits the levels of variables that augmented the contents of EPA, DHA, and EPA + DHA (% of total FAs) (see Part b). Through multiple response optimization, the predicted maximum contents of EPA (15.93%), DHA (56.37%), and EPA + DHA (72.43%) were obtained at the following process conditions: 5.17 (urea–FA ratio), −9.1 °C (crystallization temperature), and 23.37 h (crystallization time). Here, the optimization process achieved a maximum desirability of 0.907 (on a scale of 0 to 1) (Table 4, Part b). Figure 2B displays the contour plot of the estimated response surface of the urea-to-FA ratios and the temperature of crystallization. It can be deduced that the most suitable conditions to achieve high amounts of EPA and DHA should involve a relatively high urea-to-FA ratio, a prolonged crystallization time, and a low crystallization temperature. Table 4 (Part c) displays the validation of the optimized process after experimentally conducting the measurements at the same process conditions. A comparison of the predicted and experimentally obtained values indicated that both values were approximately similar for the three response surfaces (i.e., EPA, DHA, and EPA + DHA) (Table 4, Part c). In the experimental validation, a 70.43% EPA + DHA content was obtained under the following conditions: 5.0 (urea-to-FA ratio), −9 °C (the temperature of crystallization), and 24 h (the time of crystallization). This is in agreement with a previous study reporting on a stationary point of 80.51% for the value of the EPA + DHA content [20].

### 2.6. FA Composition of Optimized ω-3 PUFA Concentrates after Validation

The FA composition of the crude fish oil and the optimized ω-3 PUFA concentrates are presented in Table 1. Based on the RSM analysis, the total EPA + DHA content was raised 3.7-fold from the primary value of 18.71% in the crude oil to 70.43% in the ω-3 PUFA concentrates after being validated with the optimized process parameters (Table 4). Compared to the FA profile of the crude kilka oil in this study (Table 1), the dominant FAs found in the optimum ω-3 PUFA concentrate were DHA (58.36%), EPA (12.07%), and linoleic acid (C18:2n-6, 9.63%). The urea complexation process led to a marked decrease in the saturated (SFA) (including palmitic (C16:0) and stearic (C18:0) acids) and monounsaturated (MUFA) (including palmitoleic (C16:1n-7) and oleic (C18:1n-9) acids) contents (Table 2). The comparison of the FA groups in the crude oil used in this study and the optimized ω-3 PUFA concentrates (Table 1) showed remarkable differences in the contents of SFAs (25.6 vs. 6.5%), MUFAs (40.72 vs. 7.5%), PUFAs (24.47 vs. 80.66%), ω-3 PUFAs (20.35 vs. 70.66%), and the content of the binary EPA + DHA mixture (18.71 vs. 70.43%). Previous studies reported similar scores that ranged from 70 to 90% for ω-3 PUFA concentrates from rainbow trout [18; 74.31%], Asian catfish by-product oil [21; 88.26%], and refined salmon oil [20; 87.21%].

## 3. Materials and Methods

### 3.1. Materials

Boron trifluoride (BF3), potassium hydroxide (KOH), sodium hydroxide (NaOH), sodium sulfate anhydrous (Na_2_SO_4_), and alcalase enzyme (≥2.4 U/g) were supplied by Sigma-Aldrich (St. Louis, MO, USA). Other solvents, such as urea, n-hexane, acetone, ethanol, methanol, chloroform, acetic acid, and hydrochloric acid, were obtained from Merck (Darmstadt, Germany). The FAME standard (GLC 68D) [23] was acquired from Nu-Chek-Prep (Elysian, MN, USA).

### 3.2. Enzymatic Extraction of Fish Oil

Kilka (*C. cultriventris* caspia) fish were supplied from the Babolsar fishing harbor (Mazandaran province, Iran). They were transferred to the laboratory in iced conditions and kept at −20 °C until use. The thawed fish (50 g) were minced and then blended with distilled water (1:2, *w*/*v*) and homogenized for about 2 min at 13,000 rpm using an Ultra-Turrax (IKA-T25, Germany) digital homogenizer. The enzymatic hydrolysis was initiated by adding 1 wt% alcalase to the homogenates under optimum conditions (55 °C; pH 8.5), and the mixture was then put in an incubator shaker with agitation at 200 rpm for 4 h. Afterwards, the enzyme was inactivated by heat (10 min, 90 °C), and the mixture was centrifuged (7000× *g*, 20 min) [24]. The oil layer was separated from the supernatant and stored in tubes at −20 °C until use.

### 3.3. Derivatization of the Extracted Oil to Fatty Acid Methyl Esters (FAMEs)

At first, for the alkaline hydrolysis, about 50 mg of the obtained oil was put into tubes. Then, a NaOH/methanol solution (2 mL, 0.5 M) was added to each sample, and the obtained mixtures were heated under a reflux condenser in a hot water bath for 10 min. After cooling, 2.2 mL of a 12% (*v*/*v*) BF3 solution was added to each tube, followed by a 10 min heating at 60 °C. For obtaining a phase separation, a saturated NaCl solution (30%, *w*/*v*) was added, and n-hexane was used as the extracting solvent [25].

### 3.4. Gas Chromatography (GC) Analysis of FAMEs

FAMEs were prepared for GC using BF3-methanol reagent according to the procedure of Metcalfe et al. [26]. About 150 mg of oil was blended with 4.0 mL of a methanolic solution of NaOH (0.5 M) and warmed in a bath at 100 °C for 5 min. Next, 5.0 mL of a 12% methanolic solution of BF3 was added and the mixture was warmed for 30 min. Phase separation was obtained with 5.0 mL of a saturated sodium chloride solution using hexane as an extracting solvent (dried on anhydrous Na_2_SO_4_ before injection). GC analyses were performed on a Unicam 4600 GC (Cambridge, UK) device supplied with an FID detector in a BPX 70 column (30 m × 0.25 mm, *d*_f_ 0.22 μm). The temperature of the column was set at 160 °C for 5 min, which was followed by a temperature increase to 180 °C at 20 °C/min, and this was maintained at 180 °C for 9 min. Next, the temperature was elevated to 190 °C at 1 °C/min and maintained for 10 min. The detector and injector temperatures were 240 and 280 °C, respectively. Helium was applied as the GC carrier. The FAMEs were identified by comparing the retention times of the sample peaks with standards and were quantified as the percentage area of each FAME. The Varian Star Chromatography software (Ver. 6.41) was used for the calculation of the peak areas.

### 3.5. Production of ω-3 PUFA Concentrates via Urea Adduction

#### 3.5.1. Production of FFAs from Fish Oil

The following procedure was employed for producing FFAs from the fish oil: a 95% (*v*/*v*) aqueous ethanol solution (56 mL) was mixed with a 30% potassium hydroxide solution (34 mL). The obtained solution was added to 10 g of fish oil, and the mixture was refluxed for 2 h. To dilute the saponified mixture, distilled water (50 mL) was used, and the non-saponified fraction was removed through extraction with n-hexane (2 × 40 mL) and discarded. The obtained aqueous fraction was mixed with a 3N HCl solution; then, the FFAs were consequently extracted with n-hexane. Anhydrous sodium sulfate was used to dry the n-hexane extract, and the FFAs were recovered by evaporation of the organic solvent [22].

#### 3.5.2. The Urea Complexation Procedure

The extracted FFAs were blended with 10% (*w*/*v*) urea and a 95% (*v*/*v*) aqueous ethanol solution. The solution of FFAs was then warmed at 60 °C under magnetic stirring until a transparent solution was made. To investigate the effect of urea, the complexation was conducted at the following 5 urea–FA ratios: 2:1, 3:1, 4:1, 5:1, and 6:1. Firstly, the urea–FA mixture was allowed to crystallize at different temperatures (−24, −16, −8, 0, and 8 °C), and the crystallization was carried out at different time points (8, 16, 24, 32, and 40 h). The obtained urea–FA crystals, also known as UCF (urea complexation fraction), were isolated from the liquid fraction (known as NUCF) through filtration. The filtrate (NUCF) was diluted with an equal quantity of water and treated with a 6N HCl solution to make it acidic (pH: 4–5). The obtained solution was washed with an equal volume of n-hexane for the extraction of FFAs. The organic phase (n-hexane layer, including the released FFAs) was isolated from the aqueous phase, including urea. In order to remove the remaining urea, the last phase was repeatedly rinsed with water and then dried using sodium sulfate. Next, the solvent was evaporated by a vacuum rotary evaporator at 40 °C [21]. GC analysis was performed for determining the FA composition.

### 3.6. Procedure Optimization for Production of High-ω-3 PUFA Concentrates

A three-factor CCRD (central composite rotatable design) with 3 numeric factors at 5 levels was employed according to the RSM. It involved 20 experimental runs, involving 6 repetitions of the central point (Table 2). In this study, the urea-to-FA ratio (variable A: 2 to 6, *w*/*w*), the temperature of crystallization (variable B: −24 to 8 °C), and the time of crystallization (variable C: 8 to 40 h) were the three independent variables (Table 2). The levels of the independent variables were chosen based on the results of preliminary experiments and previous studies [10,22]. Based on the NUCF, the following response variables (R variables) were chosen: %EPA (variable R1), %DHA (variable R2), and %EPA + DHA (variable R3). To determine the experimental error, 4 replications were conducted at the central point of the experimental design. To reduce the impact of unexplained variabilities in the responses arising from parameters, all experiments were randomly performed. For the prediction of individual *Y* variables, a quadratic polynomial regression model was applied. Here, the desirability scores ranged from 0 to 1 [27]. The following equation was applied as a proposed model for each response of *Y* value:

$$Y=\beta_{0}+\sum \beta_{i\;}X_{i}+\sum \beta_{ii}X_{i}^{2}+\sum \sum \beta_{ij}X_{i}X_{ij}$$

where *β*_0_, *β*_i_, *β_ii_*, and *β_ij_* represent the intercept, linear, quadratic, and interaction regression coefficient terms, respectively, and *X_i_* and *X_j_* are independent variables.

### 3.7. Statistical Analysis

Design-Expert v. 11 (Stat-Ease, Minneapolis, MN, USA) was applied for the multiple linear regression analysis, ANOVA, and the ridge analysis of the RSREG process. Response surfaces and contour plots were generated by fitting the quadratic polynomial equation acquired from the RSREG analysis, keeping the process variables with minimal influence on the response and varying the other two variables at a fixed level. Also, by comparing the variability in the residuals of the current model with the variability among the observations in repeating the factor settings, the lack-of-fit test was performed. Also, in order to identify outliers, the method of examining the residuals, which are the difference between the observed values and the values predicted by the model, was used. Removal of outliers resulted in improved R^2^ coefficient and *p* values [28].

## 4. Conclusions

Despite its richness with proteins and oil, only a small part (~5%) of kilka caught (19,000 metric tons in 2022) from the southern coast of the Caspian Sea (Iran) is used for human consumption, and the rest is converted into fish meal and fish oil to be used as food additives in animal feed and aquafeeds. Better use of this low-cost and nutrient-rich fish can be achieved via highly promising innovative technologies focusing on the production of high-added-value biocompounds such as PUFAs. In this study, we reported on the effects of the main urea complexation process variables, and process optimization for maximizing the concentrations of ω-3 PUFAs was conducted by employing the most used variables. Here, RSM was well employed for optimizing the most important process variables in urea adduction and for gaining insight into the optimal process conditions for obtaining ω-3 PUFA concentrates with the maximum EPA and DHA contents. In this study, the urea-to-FA ratio and the temperature of crystallization showed a significant influence on modulating the EPA and DHA contents in the produced ω-3 PUFA concentrates. Our findings indicated a substantial increase in the EPA and DHA concentrations (up to 2.2 and 4.4 times, respectively) in these ω-3 PUFA concentrates produced from kilka oil; the values of both ω-3 PUFAs were 12.07 and 58.36%, respectively. After validating the analysis, our results demonstrate the feasibility of maximizing the EPA/DHA amounts in ω-3 PUFA concentrates through the optimization of the main urea adduction process variables, which confirms the hypothesis that was suggested in the ‘Introduction’. It can be concluded that urea complexation appears to be a promising approach for the separation of PUFAs from kilka oil in the food industry. Therefore, this study opens an opportunity to produce ω-3 PUFA concentrates with high concentrations of EPA + DHA (more than 70%) as FFAs and to use them in the development of new functional food and nutraceutical products enriched with ω-3 PUFAs.

## Figures and Tables

**Table 1:** Fatty acid compositions (in area percentages) of the crude kilka oil and the optimized ω-3 PUFA concentrates after validation (mean value ± SD).

Fatty Acid	Crude Oil	Optimized Concentrate
Myristic acid (C14:0)	2.57 ± 0.2	1.85 ± 0.88
Palmitic acid (C16:0)	18.23 ± 1.48	1.15 ± 0.33
Stearic acid (C18:0)	3.70 ± 0.95	1.54 ± 0.64
Behenic acid (C22:0)	1.10 ± 0.28	1.96 ± 0.44
∑SFAs	25.60	6.50
Palmitoleic acid (C16:1n-7)	4.07 ± 0.33	2.32 ± 0.80
Oleic acid (C18:1n-9)	33.47 ± 2.28	2.32 ± 0.84
Eicosenoic acid (C20:1n-9)	1.98 ± 0.21	1.11 ± 0.16
Erucic acid (C22:1n-9)	1.20 ± 0.55	1.75 ± 0.43
∑MUFAs	40.72	12.02
Linoleic acid (C18:2n-6)	2.65 ± 0.21	9.63 ± 1.14
α-Linolenic acid (C18:3n-3)/ALA	1.64 ± 0.21	0.23 ± 0.02
Arachidonic acid (C20:4n-6)	1.47 ± 0.17	0.37 ± 0.04
Eicosapentaenoic acid (C20:5n-3)/EPA	5.39 ± 0.20	12.07 ± 0.85
Docosahexaenoic acid (C22:6n-3)/DHA	13.32 ± 0.56	58.36 ± 4.04
∑PUFAs	24.47	80.66
∑EPA + DHA	18.71	70.43
∑ω-3 PUFAs	20.35	70.66

SFAs, saturated fatty acids; MUFAs, monounsaturated fatty acids; PUFAs, polyunsaturated fatty acids.

**Table 2:** Central composite design arrangement and responses for enrichment of EPA and DHA in PUFA concentrates produced from kilka oil.

Run		Variable Levels			Responses	
	A: Urea–FA (*w*/*w*)	B: Temperature (°C)	C: Time (h)	R1: EPA (%)	R2: DHA (%)	R3: EPA + DHA (%)
1	4	−8	40	14.67	57.32 ^d^	71.99 ^g^
2	3	−16	16	10.5	41.27 ^e^	51.77
3	4	−24	24	12.76	59.67	72.43
4	4	−8	24	15.37	56.35 ^f^	71.72
5	5	−16	32	15.4	51.3	66.7
6	3	−16	32	12.59	38.72	51.31
7	4	−8	24	15.71	50.8	66.51
8	5	−16	16	12.6	58.05	70.65
9	4	−8	24	17.32	50.8	68.12
10	4	−8	24	17	46.47	63.47
11	4	−8	8	9.86	44.11	53.97
12	5	0	32	16.15	49.97	66.12
13	3	0	16	12.14	37.26	49.4
14	5	0	16	15.61	54.17	69.78
15	4	−8	24	16.67	49.36	66.03
16	4	8	24	12.33 ^a^	42.51	54.84
17	4	−8	24	16.56	51.2	67.76
18	3	0	32	15.41	36.63	52.04
19	2	−8	24	0 ^b^	40.63	40.63
20	6	−8	24	9.57 ^c^	58.6	68.17

^a, b, c, d, e, f, g^ These data were detected as outliers by the model and ignored in the process so that the results of the model predictions would have better accuracy.

**Table 3:** Regression coefficients and *p*-values of predictive second-order polynomial models for each of the response variables.

Process Variables ^a^			Response Variables			
	R1 (%EPA)		R2 (%DHA)		R3 (%EPA + DHA)	
	Coefficient	*p* Value	Coefficient	*p* Value	Coefficient	*p* Value
Intercept	16.44		49.77		67.3	
Linear						
A	1.14	0	5.27	0	7.74	0
B	1.03	0.01	−3.56	0	−2.39	0.02
C	1.15	0	−3.11	0.01	−0.63	0.61
Quadratic						
A*A	−1.19	0.03	−1	0.99	−3.2	0
B*B	−0.4	0.15	0.36	0.53	−0.89	0.24
C*C	−1.04	0	−2.91	0.01	−3.59	0.01
Interaction						
A*B	−0.09	0.76	1.52	0.2	0.02	0.98
A*C	−0.25	0.39	0.44	0.7	−1.22	0.36
B*C	−0.13	0.64	1.97	0.12	0.42	0.74
Lack of fit		0.34		0.17		0.16
R^2^	0.95			0.94		0.93
Adjusted R^2^	0.8818			0.8731		0.8543

R^2^, regression coefficient. ^a^ Process variable (A, B, and C) as expressed in Table 2.

**Table 4:** Optimization of the process variables and the multiple response variables.

Part (a) Optimization of Process Variables					
Independent Variables		Process Variables ^a^		Stationary Point	Optimum Value ^b^
	A	B	C		
EPA	4.67	1.62	27.48	Maximum	17.4
DHA	5.76	−13.35	20.92	Maximum	60.76
EPA + DHA	5.31	−13.97	21.52	Maximum	73.66
**Part (b) Multiple Response Optimization of Response Variables**					
**Independent Variables**		**Process Variables**		**Stationary Point**	**Predicted Value ^b^**
	A	B	C		
EPA					15.93
DHA	5.17	−9.1	23.37	Maximum	56.37
EPA + DHA					72.43
Maximum desirability					0.907
**Part (c) Experimental Validation of the Multiple Response Optimization of the Dependent Variables**					
**Independent Variables**		**Process Variables**		**Stationary Point**	**Experimental Value ^b^**
	A	B	C		
EPA					12.07
DHA	5	−9	24	Maximum	58.36
EPA + DHA					70.43

^a^ Process variables (A, B, and C) as expressed in Table 2. ^b^ Values expressed as % of total FAs.

## Data Availability

Data are contained within the article.
